# The relationship between elevated magnesium levels and coronary artery ectasia

**DOI:** 10.5830/CVJA-2016-023

**Published:** 2016

**Authors:** Yolcu Mustafa, Ipek Emrah, Yıldırım Erkan, Rifat Ulusoy Fatih, Turkmen Serdar, Sertcelik Alper, Ozen Yücel

**Affiliations:** Department of Cardiology, Arel Universty, Private Medicana Camlica Hospital, Istanbul, Turkey; Department of Cardiology, Erzurum Region Training and Research Hospital, Erzurum, Turkey; Department of Cardiology, Erzurum Region Training and Research Hospital, Erzurum, Turkey; Department of Cardiology, Erzurum Region Training and Research Hospital, Erzurum, Turkey; Department of Cardiology, Sani Konukoğlu Medical Centre, Gaziantep, Turkey; Department of Cardiology, Sani Konukoğlu Medical Centre, Gaziantep, Turkey; Department of Cardiovascular Surgery, Kartal Kosuyolu High Specialty Education and Research Hospital, Erzurum, Turkey

**Keywords:** coronary artery ectasia, magnessium, pathophysiology

## Abstract

**Backround:**

Coronary artery ectasia (CAE) without specific symptoms is the localised or diffuse swelling of the epicardial coronary arteries. Magnessium (Mg) plays an important role in cardiac excitability, vascular tonus, contractibility, reactivity and vasodilatation. In our research, we aimed to study the vasodilatory effect of Mg in the aetiopathogenesis of ectasia.

**Methods:**

Patients identified during routine coronary angiograms in our clinic between January 2010 and 2013 were included in the study. Sixty-two patients with isolated CAE, 57 with normal coronary angiograms (NCA), 73 with severe coronary artery disease (CAD), and 95 with stenosis of at least one coronary artery and CAE (CAD + CAE) were included in the study. Serum Mg levels were measured in mg/ dl after 12 hours of fasting.

**Results:**

There were no statistically significant differences between the groups in terms of age, hypertension, smoking, hyperlipidaemia, diabetes mellitus, family history of coronary artery disease and medications used. Serum glucose, thyroid stimulating hormone (TSH), urea, total cholesterol, triglyceride, low-density lipoprotein (LDL) cholesterol, sodium and potassium levels were similar in all groups. Serum Mg levels were 1.90 ± 0.19 mg/dl in patients with isolated CAE, 1.75 ± 0.19 mg/dl in those with CAD, 1.83 ± 0.20 mg/dl in those with CAD + CAE, and 1.80 ± 0.16 mg/dl in the NCA group. These results show that Mg levels were higher in ectasia patients with or without CAD.

**Conclusions:**

The histopathological characteristics of patients with CAE were similar to those with CAD. The specific mechanism of abnormal luminal dilatation seen in CAE however remains to be elucidated. Mg is a divalent cation with powerful vasodilatory effects. In our study, serum Mg levels were found to be statistically higher in ectasia patients with or without CAD.

## Backround

Coronary artery ectasia (CAE) without specific symptoms is the localised or diffuse swelling of the epicardial coronary arteries to at least 1.5 times the adjacent normal coronary segment.^1^,^2^ It is congenital or acquired and several studies have reported its incidence at 0.3–5%.^1^,^3^

Atherosclerosis, congenital factors, and inflammatory or connective tissue disorders may play a role in the aetiology, however, the aetiopathogenesis remains unclear despite some molecular, cellular and vascular mechanisms being defined in previous studies.^4^,^5^ In several studies, other vascular structures were shown to be involved in CAE patients, which indicates CAE is a vascular disease and not localised to the coronary arteries. Therefore factors other than atheroscleosis may play a role in its aetiopathogenesis.

Magnesium, the second most abundant intracellular cation, is an essential element that plays a crucial role in cardiac and vascular functions. Magnesium regulates contractile proteins, modulates transmembrane transport of calcium (Ca2+), sodium (Na+) and potassium (K+), acts as a co-factor in the activation of ATPase, controls regulation of energy-dependent cytoplasmic and mitochondrial metabolism, and influences DNA and protein synthesis at the subcellular level.^6^,^7^6,7

Small changes in concentration of extracellular and/or intracellular free Mg have important effects in cardiac excitability, vascular tonus, contractibility, reactivity and growth.^8^,^9^ Low levels of intracellular Mg lead to abnormal vascular cell growth, inflammation, fibrosis and contraction, resulting in positive vascular remodelling. Dosing with Mg was found to cause vasodilatation and to have anti-inflammatory effects.^8^,^9^

In our study, we aimed to study the vasodilatory effect of Mg in the aetiopathogenesis of ectasia, and the long-term effects of elevated Mg levels on the vascular structure, leading to abnormal coronary dilatation.

## Methods

A total of 4 800 patients identified during routine coronary angiograms in our clinic between January 2010 and 2013 were included in the study. The study was planned to be prospective and was approved by the local ethics committee. After coronary angiography, the patients were informed about the study and written consents were given.

Sixty-two patients with isolated CAE, 57 with normal coronary angiograms (NCA), 73 with severe coronary artery disease (CAD), and 95 with stenosis of at least one coronary artery and CAE (CAD + CAE) were included in the study. All of the patients were questioned on their cardiovascular risk factors and medication used. Routine biochemical and haematological laboratory tests were done.

Previous history of myocardial infarction, percutaneous coronary intervention, left ventricular hypertrophy, left ventricular dysfunction [ejection fraction (EF) < 50%], moderate to severe valvular disease, rhythms other than sinus, congenital heart disease, chronic obstructive lung disease and/or cor pulmonale, chronic systemic illness, active infection, renal failure, neoplastic disease, antioxidant drug usage and alcohol abuse were the exclusion criteria.

Coronary angiography was performed on a Siemens Axiom Artis angiography device with standard Seldinger’s technique using isohexol. In order to evaluate each coronary artery, at least four views from the left and two views from the right side were taken. Patients were allocated into four groups: patients with CAD, those with isolated CAE, those with CAD + CAE, and subjects with normal coronary angiograms. Angiographic images were evaluated by two independent researchers.

Isolated CAE was defined as dilatation of at least one epicardial coronary artery to 1.5 times the reference vessel diameter and absence of critical stenosis (> 50%) in any of the coronary arteries. NCA were defined as the absence of angiographic atherosclerosis during routine coronary angiography; 60% or greater stenosis in at least one epicardial coronary artery was defined as CAD. CAD + CAE was defined as 60% or greater stenosis in at least one epicardial coronary artery and the presence of ectasia in any of the coronary arteries.

Serum Mg levels were measured in mg/dl after 12 hours of fasting. Haemograms, renal and liver function tests, lipid profiles, serum glucose and electrolytes and thyroid stimulating hormone (TSH) levels were also evaluated in all patients.

## Statistical analysis

Statistical analysis was performed using the SPSS 14 (SPSS Inc, Chicago, IL, USA) statistics program. Data are given as percentages and mean ± standard deviation. ANOVA and post hoc Tukey tests were used in the comparison of parametric variables between groups. A chi-squared test was performed in the comparison of non-parametric values and percentages. Statistical significance level was taken as p ≤ 0.05.

## Results

The mean age was 62 ± 10 years in the CAE patients, 61 ± 11 years in CAD patients, 64 ± 8 years in those with CEA + CAD, and 59 ± 8 years in the NCA patients. There was no statistically significant difference between the groups in terms of age, hypertension, smoking, hyperlipidaemia, diabetes mellitus, family history of CAD, and medications used (Table 1).

**Table 1:** Comparisons of cardiovascular risk factors and the medications used

*Parameters*	*Isolated CAE (n = 62)*	*CAD (n = 73)*	*CAD + CAE (n = 95)*	*NCA (n = 57)*	*p-value*
Age (years)	62 ± 10	61 ± 11	64 ± 8	59 ± 8	0.062
Gender (M/F)	45/17	39/34	76/19	17/40	0.000
Hypertension (n)	43	56	74	35	0.119
Diabetes mellitus (n)	13	21	25	12	0.648
Hyperlipidaemia (n)	11	13	29	9	0.080
Family history of CAD (n)	18	25	36	16	0.543
Smoking (n)	33	32	49	24	0.482
Calcium channel blockers (n)	12	20	23	21	0.744
Beta-blockers (n)	24	20	38	12	0.051
Angiotensin converting enzyme inhibitors (n)	21	16	33	11	0.084
Angiotensin receptor blockers (n)	17	26	32	14	0.471
Diuretics (n)	15	22	40	16	0.084
Oral antidiabetic (n)	11	13	16	10	0.998

Serum glucose, calcium, TSH, urea, total cholesterol, triglycerides, low-density lipoprotein (LDL) cholesterol, sodium and potassium levels were similar in both groups (Table 2). Serum creatinine level was within normal limits in all patients, however creatinine values were statistically lower in the NCA group (p = 0.024). High-density lipoprotein (HDL) cholesterol levels were lowest in the CAD patients and highest in the isolated ectasia group, and this difference was statistically significant (p = 0.003).

**Table 2:** Comparisons of clinical parameters and magnessium

*Clinical parameters*	*Isolated CAE (n = 62)*	*CAD (n = 73)*	*CAD + CAE (n = 95)*	*NCA (n = 57)*	*p-value*
Fasting blood glucose (mg/dl)	109 ± 28	113 ± 25	118 ± 39	108 ± 25	0.197
(mmol/l)	(6.05 ± 1.55)	(6.27 ± 1.39)	(6.55 ± 2.16)	(5.99 ± 1.39)	
Urea (mg/dl)	35 ± 10	35 ± 10	36 ± 10	32 ± 10	0.114
Serum creatinine (mg/dl)	0.91 ± 0.20	0.88 ± 0.22	0.92 ± 0.31	0.80 ± 0.15	0.024^¥^
(mmol/l)	(80.44 ± 17.68)	(77.79 ± 19.45)	(81.33 ± 27.40)	(70.72 ± 13.26)	
Sodium (mg/dl)	139 ± 2.09	139 ± 2.67	140 ± 2.24	139 ± 2.60	0.102
Potassium (mEq/l)	4.32 ± 0.43	4.17 ± 0.42	4.28 ± 0.43	4.12 ± 0.57	0.054
Total cholesterol (mg/dl)	192 ± 43	188 ± 35	180 ± 39	179 ± 36	0.142
(mmol/l)	(4.97 ± 1.11)	(4.87 ± 0.91)	(4.66 ± 1.01)	(4.64 ± 0.93)	
Triglycerides (mg/dl)	146 ± 79	171 ± 112	151 ± 82	136 ± 59	0.133
(mmol/l)	(1.65 ± 0.89)	(1.93 ± 1.27)	(1.71 ± 0.93)	(1.54 ± 0.67)	
HDL cholesterol (mg/dl)	46 ± 12	39 ± 8.8	42 ± 9.5	44 ± 10	0.003^μ^^√^
(mmol/l)	(1.19 ± 0.31)	(1.01 ± 0.23)	(1.09 ± 0.25)	(1.14 ± 0.26)	
LDL cholesterol (mg/dl)	118 ± 36	114 ± 27	108 ± 34	105 ± 29	0.095
(mmol/l)	(3.06 ± 0.93)	(2.95 ± 0.70)	(2.80 ± 0.88)	(2.72 ± 0.75)	
TSH (mIU/l)	0.99 ± 0.87	1.10 ± 1.45	1.12 ± 1.21	1.16 ± 1.35	0.895
Magnesium (mg/dl)	1.90 ± 0.19	1.75 ± 0.19	1.75 ± 0.19	1.80 ± 0.16	0.000^√^^£^^&^
Calcium (mg/dl)	9.69 ± 0.32	9.64 ± 0.28	9.67 ± 0.33	9.58 ± 0.41	0.103

^¥^ NCA vs CAD + CAE (p < 0.05); ^μ^CAD vs NCA (p < 0.05); ^√^CAD vs CAE (p < 0.05); ^£^CAE vs NCA (p < 0.05); ^&^CAD vs CAD + CAE (p < 0.05).

Serum Mg levels were 1.90 ± 0.19 mg/dl in isolated CAE patients, 1.75 ± 0.19 mg/dl in those with CAD, 1.83 ± 0.20 mg/ dl in those with CAD + CAE, and 1.80 ± 0.16 mg/dl in the NCA group. These results showed that Mg levels were higher in the ectasia patients with or without CAD.

## Discussion

Mg is a divalent cation with powerful vasodilatory effects. In our study, serum Mg levels were found to be statistically higher in the ectasia patients with or without CAD.

CAE is dilatation of the coronary arteries to at least 1.5 times normal, and the basic pathogenic mechanism is destruction of the musculo-elastic layers of the arterial tunica media, and the accumulation of collagen in place of elastin, leading to thinning of the arterial wall.^1^,^5^,^10^ Injury of the media causes decreased stress tolerance of the vessel wall to intraluminar pressure, leading to progressive dilatation and ectasia formation.^10^,^11^ In a pathological examination, atherosclerosis is detected in more than 50% of patients, however connective tissue disorders and vasculitides can also be present.^11^

CAE can be divided into four different types according to the classification of Markis and colleagues. Type 1 indicates diffuse ectasia in two to three different vessels, type 2 shows diffuse disease in one vessel and local disease in another, type 3 is diffuse disease in one vessel, and type 4 indicates localised or segmental ectasia. In our study there were 24 (16%) patients with type 1 CAE, 43 (27%) with type 2, 54 (34%) with type 3, and 36 (23%) with type 4 CAE.

The histopathological characteristics of CAE are similar to those of CAD, however the specific mechanism of abnormal luminal dilatation seen in CAE remains to be elucidated. Negative remodelling is found in stenotic CAD, however positive remodelling is seen in CAE.^12^ In a study by Yolcu and colleagues, it was shown that serum levels of plasminogen activator inhibitor-1, which causes an increase in activity of matrix metalloproteinase, increased in patients with isolated ectasia, suggesting different pathways other than atherosclerosis in ectasia formation.^12^

Yetkin and colleagues showed that carotid–intima media thickness was statistically lower in CAE patients with stenotic CAD than in individuals who had CAD alone, and reported that ectasia was not an atherosclerotic process limited to the coronary arteries.^13^ In previous studies, aortic aneursym, dilatations in lower-extremity varicose veins, basillary artery aneurysm and varicocele were reported to be more frequent in isolated ectasia patients.^12^ These findings propose that positive remodelling in the vessel wall, which is not common in the atherosclerotic process, plays a role in the aetiopathogenesis of CAE.

Mg^2+^, which works as an allosteric modulator of several proteins, controls nucleotide and protein synthesis, regulates Na^+^, K^+^, and Ca2^+^ channels, and plays a crucial role in enzymatic reactions involving kinases, is an abundant intracellular divalent cation.^14^,^15^ Less than 1% of the total body Mg2^+^ concentration circulates in the blood, and it is stored primarily in bone and the intracellular compartments of muscle and soft tissue.^15^,^16^15,16 Mg2^+^ regulates vascular tone, cardiac rhythm and platelet-activated thrombosis.^17^,^18^

Mg stimulates nitric oxide release, which has a potent vasodilatory effect, from the endothelium. It is a co-factor for the delta-6-desaturase enzyme, which plays an important role in the synthesis of prostoglandin E1 (it has vasodilatory and antiplatelet effects) from linoleic acid.^19^

An increase in extracellular Mg concentration causes vasodilatation, a reduction in vascular resistance, an increase in capacitance function in peripheral, coronary and cerebral arteries, and a decrease in agonist-induced vasoconstriction. Mg deficiency causes oxidative stress, inflammation, decreased luminal diameter, medial hypertrophy, vascular remodelling, it potentiates agonist-evoked vasoconstriction, and increases vascular tonus.^20^

As a result of increased intracellular Mg2+ concentration [(Mg2^+^)i], vasodilation occurs and agonist-induced vasoconstriction decreases. Reduced (Mg2^+^)i leads to hypercontractility and it impairs vasorelaxation.^21^

Mg is a unique calcium antagonist, has an effect on most types of calcium channels in vascular smooth muscle, and can decrease intracellular calcium levels.^22^ Inactivation of calmodulin-dependent myosin light-chain kinase activity and decreased contraction are among the major effects of decreased intracellular calcium levels.^22^ Consequently, this causes arterial relaxation, lower peripheral and cerebral vascular resistance, it relieves vasospasm, and results in a decline in arterial blood pressure.^22^

As a calcium antagonist, Mg decreases the activity of voltagedependent calcium channels, diminishing calcium release from the sarcoplasmic reticulum.^23^ In some in vivo and in vitro studies, Mg was shown to have vasodilatory effects on the aorta, and mesenteric, skeletal muscular, uterine and cerebral arteries.^23^

In previous studies, Mg was reported to play a role in the aetiopathogenesis and management of eclampsia and hypertension. Eclampsia is characterised by myogenic vasoconstriction of the cerebral arterioles and arteries, increased permeability of the blood–brain barrier, and oedema formation due to acute blood pressure increase.^23^ In those patients, intravenous Mg, due to its calcium antagonist effect on smooth muscle, caused relaxation and vasodilatation.^23^ It also limits vasogenic oedema in cerebral endothelium by a calciumdependent secondary messenger system, leading to decreased paracellular permeability and stress fibre contraction.^23^

Mg is now being used in coronary stents because of its strong antiproliferative and vasodilatory effects. In a study by Yener and colleagues, it was shown that Mg supplementation after coronary artery bypass surgery may delay the onset of atrial fibrillation.^24^

In studies related to the aetiopathogenesis of hypertension, a Mg deficiency was reported to have hypertensive effects, and dietary Mg intake was related to hypotension, showing the reverse positive relationship between blood pressusure and serum Mg levels.^20^ The basic mechanism at play is blood pressure regulation by Mg via modulating vascular tone and reactivity.^20^

The direct vascular effect of Mg was first suggested in early 1990 in a study in which Mg salt infusion was reported to have a blood pressure-lowering effect by decreasing peripheral vascular resistance, with a mild increase in myocardial contractility.^25^ Observational studies support these clinical findings and acute Mg infusion causes hypotension via its vasodilatory effect.^26^

Similar to previous studies, our research found CAE to be significantly more prevalent in males.^1^,^4^ Although serum creatinine levels were in the normal range in all our study groups, there was a statistically significant difference between the groups, due possibly to small differences in creatinine levels. However in the review by Cunningham and colleagues, it was reported that in the early stages of renal failure, there was no change in Mg metabolism but in the end stage, Mg levels were affected.^27^ Therefore normal creatinine levels in our study groups probably did not affect the Mg balance.^27^

In our study, there was a significant difference in HDL cholesterol levels between the groups. In a study by Randell and colleagues, HDL cholesterol levels were found to be positively correlated with Mg levels. Our results are consistent with this study, showing higher Mg and HDL cholesterol levels in isolated ectasia and lower levels in CAD patients, indicating that there was no impact of this correlation on our results.^28^

In our study, serum Mg levels were statistically higher in isolated ectasia patients than in the NCA and CAD groups. Mg levels were lowest in the CAD group. Mg levels in the CAD + CAE group were higher than in the NCA group but lower than in the isolated ectasia group. The higher levels of Mg in the CAD + CAE than in the CAD group reached statistical significance.

White blood cell count, as an indicator of inflammation, was significantly lower in isolated ectasia patients than in the CAD group, relating to the anti-inflammatory effect of Mg. Another inflammatory marker, ESR, was also found to be higher in CAD patients than in isolated ectasia patients. Mg in the extracellular fluid constitutes only 1% of the total body Mg concentration. However our findings suggest that chronically higher levels of serum Mg, with its anti-inflammatory effects, play a crucial role in the pathogenesis of ectasia by leading to vasodilation and negative remodelling. We proposed that factors other than atherosclerosis may play an important role in ectasia formation.

## Conclusion

The histopathological characteristics of patients with CAE were similar to those with CAD. The specific mechanism of abnormal luminal dilatation seen in CAE however remains to be elucidated. Mg is a divalent cation with powerful vasodilatory effects. In our study, serum Mg levels were found to be statistically higher in ectasia patients with or without CAD.
